# Microcystin-LR-Induced Interaction between M2 Tumor-Associated Macrophage and Colorectal Cancer Cell Promotes Colorectal Cancer Cell Migration through Regulating the Expression of TGF-β1 and CST3

**DOI:** 10.3390/ijms241310527

**Published:** 2023-06-23

**Authors:** Xinying Jiang, Hailing Zhang, Hengshuo Zhang, Fan Wang, Xiaochang Wang, Tong Ding, Xuxiang Zhang, Ting Wang

**Affiliations:** 1 Department of Cell Biology, School of Basic Medical Sciences, Nanjing Medical University, Nanjing 211166, China; 2 State Key Laboratory of Pollution Control and Resource Reuse, School of the Environment, Xianlin Campus, Nanjing University, Nanjing 210023, China

**Keywords:** MC-LR, TGF-β1, M2 tumor-associated macrophages, migration, colorectal cancer

## Abstract

Microcystin-LR (MC-LR) is a toxic secondary metabolite produced by cyanobacteria that has been demonstrated to promote colorectal cancer (CRC). However, the mechanism by which MC-LR enhances CRC in the tumor microenvironment (TME) is poorly understood. To elucidate its role in TME, a co-culture system was established using CRC cells and M2 macrophages in a Transwell chamber. The study found that MC-LR promotes CRC cell migration by upregulating TGF-β1 expression and secretion in M2 macrophages and downregulating CST3 in CRC cells. Neutralizing TGF-β1 increased CST3 expression in CRC cells, while overexpressing CST3 in CRC cells suppressed TGF-β1 expression in M2 macrophages, both of which weakened MC-LR-induced cellular motility in the co-culture system. In vivo, the mice in the MC-LR/AOM/DSS group had more tumor nodules, deeper tumor invasion, and higher M2 macrophage infiltration compared to the AOM/DSS group, and the expression of TGF-β1 and CST3 in tumors was consistent with the cellular level. Overall, this study provides insights into the regulatory mechanism of MC-LR on TME, revealing that MC-LR upregulates the expression and secretion of TGF-β1 in M2 macrophages, which in turn inhibits the expression of CST3 in CRC cells to promote migration.

## 1. Introduction

Microcystins are cyclic heptapeptide toxins generated by freshwater cyanobacteria, with microcystin-LR (MC-LR) being the most prevalent congeners [1]. The World Health Organization (WHO) guidelines limit MC-LR in drinking water to 1 μg/L [2]. However, field studies have shown that the concentration of microcystins in lakes or reservoirs where harmful algal blooms occur far exceeds the standard. For example, in Lake Okeechobee and the St. Lucie River estuary in Florida, the concentration of microcystins can reach over 10 μg/L [3]. In Taihu Lake in China, the concentration of microcystin can reach 6.69 mg/L in summer and 15.6 mg/L in October [4]. The presence of a high MC concentration of up to 670 μg/L was also detected in Sagar Lake, India [5]. As a result, the toxins may pose a potential threat to human health in the form of drinking water, skin contact, or the food chain. MC-LR has been shown to inhibit protein serine/threonine phosphatases 1 and 2A [2] and has been linked to several toxic effects, including liver damage, nephrotoxicity [6,7], immunotoxicity [8,9], and effects on cell morphology and cellular adhesion [10]. Furthermore, epidemiological studies suggest that chronic exposure to MC-LR can increase the incidence of various cancers, including hepatic carcinoma [11] and CRC [12], and may contribute to cancer progression [13]. Recent research has demonstrated that the tumor-promoting activity of MC-LR is associated with its capacity to enhance the expression of Nrf2 in liver cancer cells [14] and alter the structure of intestinal flora in mouse models of liver cancer, resulting in a carcinogenic form [15]. Moreover, our previous investigations have indicated that MC-LR activates the PI3-K/AKT signal pathway, leading to an epithelial–mesenchymal transition [16], and regulates the expression of miR-221/PTEN and STAT3 signaling pathways [17], thereby facilitating the invasion and metastasis of CRC cells.

The tumor microenvironment (TME) is a complex milieu where cancer cells interact with stromal cells through multiple biochemical, physical signals and cellular contact, playing a critical role in cancer progression. Macrophages are among the most abundant myeloid cells in major human solid malignancies, and they are prone to be polarized into two distinct subtypes, the classically activated macrophages (M1) and the alternatively activated macrophages (M2), in response to different environmental stimuli [18]. Tumor-associated macrophages (TAMs) are believed to more closely resemble M2-polarized macrophages [19]. Studies have shown that TAMs promote the invasion and metastasis of CRC cells through the NF-κB pathway via the secretion of IL10 and IL17 [20], and the production of matrix proteins (MMP-2/9) and proteases from M2 macrophages alters the extracellular matrix composition to promote the invasion and migration of pancreatic cancer cells [21]. Thus, TAMs play a vital role in the development, invasion, and metastasis of malignant tumors [22], immune evasion [23], and the formation of blood vessels [24] and lymphatics [25]. Clinically, the density of TAMs in different tumor tissues is significantly correlated with a poor prognosis of the tumor [20,26]. Generally, tumor cells create a local microenvironment conducive to their survival and development through autocrine and paracrine mechanisms, under which the TME promotes tumor cell invasion and metastasis by altering the metabolism, secretion, immunity, and other functions of tumor cells.

In addition to its impact on CRC cells, the role of TME cannot be overlooked in the onset, progression, recurrence, and metastasis of CRC. However, there have been limited investigations on the regulation of TME by MC-LR. Therefore, it is imperative to further examine the regulatory effect of MC-LR on TME in cancer progression. In this study, we demonstrate for the first time that MC-LR is involved in regulating the interaction between M2 macrophages and tumor cells in TME, ultimately promoting CRC cell migration. Our findings provide crucial experimental evidence for enhancing clinical outcomes and treating MC-LR-induced CRC.

## 2. Results

### 2.1. MC-LR Promoted the Migration of CRC Cells and the Change of Secreted Protein

**Figure 1:**
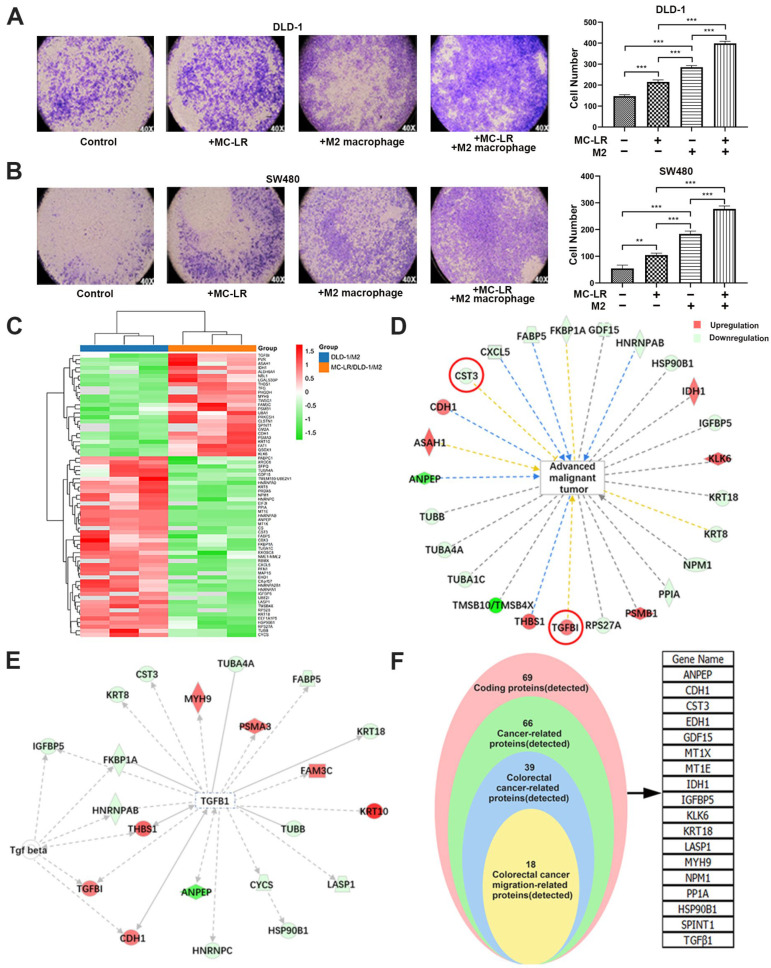
MC-LR-induced CRC cell–M2 macrophage interaction promoted CRC cell migration and analysis of relative proteins. The effect of MC-LR on the migration of (**A**) DLD-1 and (**B**) SW480 cells was determined by Transwell. The migration cells were stained with crystal violet, and the pictures were captured under light microscopy (×40). Secreted proteins were detected by iTRAQ quantitative proteomics technology. Samples were harvested from DLD-1 cell–M2 macrophage co-culture system with or without 25 nM MC-LR treatment for 48 h. (**C**) Differential expression proteins in co-culture system were colored based on the heat map scale (upregulated: red, FC ≥ 1.2; downregulated: blue, FC ≤ −1.2) by iTRAQ quantitative proteomics technology. IPA was used to analyze (**D**) the relationship between advanced malignant tumors and screened proteins, and (**E**) the interaction between TGF-β1 and related proteins. Red represents increased proteins; and green represents decreased proteins. (**F**) The proteins related to the migration of CRC cells were screened out. All data were presented as the mean ± SD from at least three independent experiments. ** *p* < 0.01, and *** *p* < 0.001, compared with the control group.

In this study, we established a co-culture system composed of M2 macrophages and CRC cells to mimic the TME formed by tumor cells and macrophages to better explore the interaction between M2 TAM and CRC cells in the presence of MC-LR. Firstly, THP-1 cells were treated with PMA to obtain M0 macrophages and then stimulated by IL4/IL13 to obtain M2 macrophages. The results of the flow cytometric assay displayed a higher ratio of CD163+ and CD206+ cells in PMA/IL4/IL13-treated THP-1 cells than PMA-treated THP-1 cells (Figure S1B,C). In addition, qPCR results showed higher mRNA levels of *IL-10*, *Arg-1*, *TGF-β1*, and *CD206* after being stimulated by IL4/IL13 (Figure S1A), indicating the polarization of the M2-like macrophage. Then, the experiment of migration was performed by Transwell to explore the effect of MC-LR in the co-culture system, and, compared with the untreated group, MC-LR could strikingly increase the number of migrated CRC cells under the co-culture system (Figure 1A,B). Subsequently, iTRAQ quantitative proteomics technology was utilized to explore the secreted proteins in the supernatant from the DLD-1-M2 macrophage co-culture system. As the results show in Figure 1, 69 significantly differential expression proteins were identified in the MC-LR treatment group, compared with the control group (Figure 1C), and a GO enrichment analysis of differential expression proteins illustrated that proteins associated with the cellular/metabolic process, extracellular regions, and binding activity were enriched in response to the exposure of MC-LR (Figure S2), which might be related to the progression of the tumor. Then, IPA analysis revealed 25 proteins linked to tumor progression (Figure 1D). Among them, TGF-β1, a marker of M2 macrophages that might affect the interaction between CRC cells and M2 macrophages, was upregulated in the MC-LR-treated group. Then, to explore secreted proteins correlated with TGF-β1 in the co-culture system, further analysis by IPA showed that CST3 demonstrated an opposite trend, which might have a strong correlation (Figure 1E). In addition, proteins associated with CRC migration were screened out, including TGF-β1 and CST3 (Figure 1F).

### 2.2. MC-LR Promoted the Expression of TGF-β1 and Reduced the Expression of CST3 in CRC Cell–M2 Macrophage Co-Culture System

**Figure 2:**
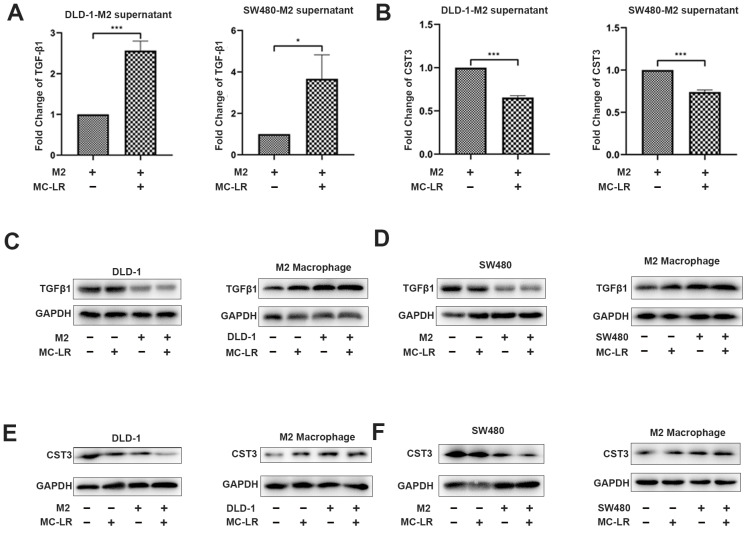
MC-LR promotes the expression of TGF-β1 in M2 macrophages and inhibits the expression of CST3 in CRC cells under the co-culture system. (**A**) Secretion of TGF-β1 was detected by ELISA in the supernatant of co-culture system with 25 nM MC-LR for 48 h. (**B**) Secretion of CST3 was detected by ELISA in the supernatant of co-culture system with 25 nM MC-LR for 48 h. Protein expression of TGF-β1 and CST3 in (**C**,**E**) DLD-1 cell–M2 macrophage and (**D**,**F**) SW480 cell–M2 macrophage co-culture system was detected by Western blotting, respectively. All data were presented as the mean ± SD from at least three independent experiments. * *p* < 0.05, and *** *p* < 0.001, compared with the control group.

Based on the above analysis, we detected the secretion of TGF-β1 in the DLD-1 cell–M2 macrophage and SW480 cell–M2 macrophage co-culture systems by ELISA. The expression of TGF-β1 in the co-culture systems increased after the exposure of MC-LR (Figure 2A). In view of the fact that secretion in co-culture system is the main interaction between cells, it is necessary to explore which cells have increased the secretion of TGF-β1. Next, Western blotting was performed to understand what kind of cells increased the secretion of TGF-β1 after the exposure of MC-LR in the co-culture system. The results demonstrated that the protein expression of TGF-β1 in M2 macrophages was enhanced in the co-culture system after the exposure of MC-LR (Figure 2C,D, lanes 3 and 4 in M2 macrophage), and the supernatant of MC-LR-treated CRC cells also promoted the expression detected by qPCR (Figure S1F,G), while the expression in CRC cells had no obvious changes (Figure 2C,D, lanes 3 and 4 in DLD-1 and SW480), suggesting that the elevated TGF-β1 induced by MC-LR in the co-culture system was mainly secreted by M2 macrophages. At the same time, the results of ELISA also showed that the secretion of CST3 was obviously inhibited in the DLD-1 cell–M2 macrophage and SW480 cell–M2 macrophage co-culture systems after the exposure of MC-LR (Figure 2B). Subsequently, Western blotting proved that the reduced mRNA and protein expression of CST3 could be attributed to CRC cells after the exposure of MC-LR in the co-culture system (Figure 2E,F, lanes 3 and 4 in DLD-1 and SW480). Interestingly, MC-LR exposure did not evidently alter the expression of CST3 in M2 macrophages in the co-culture system (Figure 2E,F, lanes 3 and 4 in M2 macrophage). The above results showed that with the condition of co-culture, MC-LR could enhance the expression of TGF-β1 in M2 macrophages and decrease the expression of CST3 in CRC cells. Next, further study for whether the changes could account for the promoted migration of CRC cells in co-culture systems was conducted.

### 2.3. The Expression of TGF-β1 and CST3 in Co-Culture System Regulated by MC-LR Prompted the Migration of CRC Cells

**Figure 3:**
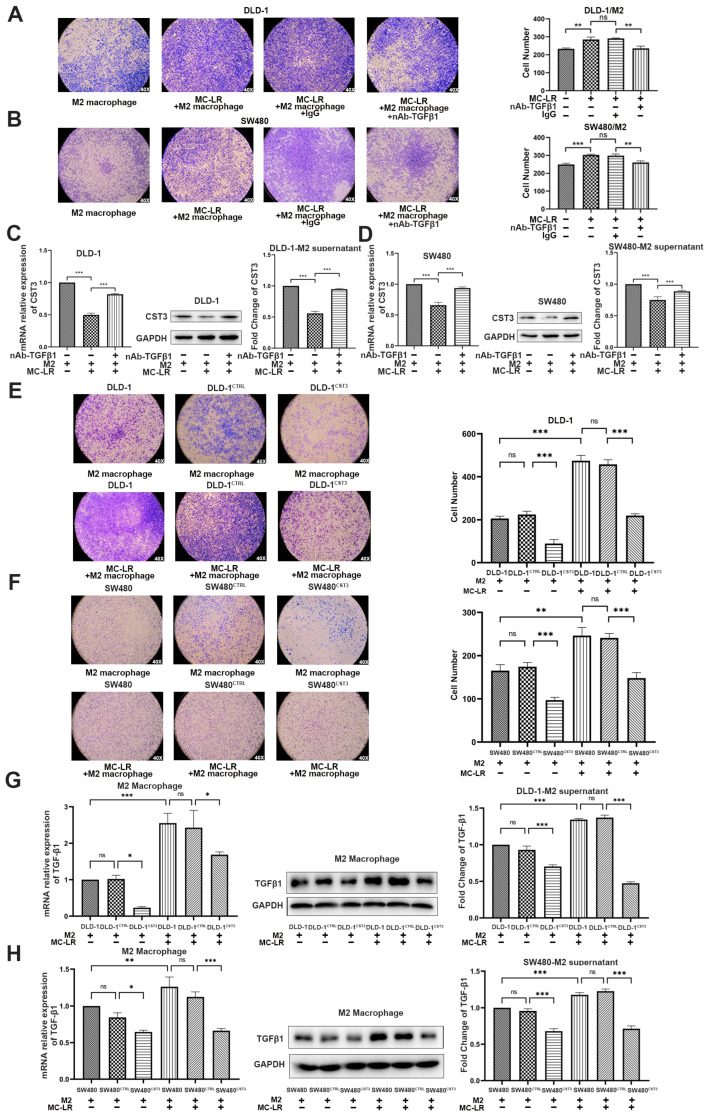
MC-LR prompted CRC cell migration by regulating the expression of TGF-β1 and CST3 in the co-culture system. The migration of (**A**,**B**) CRC cells in the co-culture system. Migration of (**A**) DLD-1 cells and (**B**) SW480 cells in the co-culture system which were treated with 0.8 μg/mL TGF-β1-neutralizing antibody and 25 nM MC-LR. IgG was used as a negative control. The pictures were captured under light microscopy (×40). mRNA and protein expression and secretion of CST3 in (**C**) DLD-1 cell and (**D**) SW480 cells -M2 macrophage co-culture systems which were treated with 25 nM MC-LR or 25 nM MC-LR/TGF-β1-neutralizing antibody (0.8 μg/mL) for 48 h, detected by qPCR, Western blotting, and ELISA, respectively. The migration of (**E**) DLD-1 and (**F**) SW480 cells with overexpressing CST3 which were co-cultured with M2 macrophages and MC-LR is at the concentration of 25 nM for 48 h. The pictures were captured under light microscopy (×40). mRNA and protein expression and secretion of TGF-β1 in M2 macrophages in (**G**) DLD-1 cells and (**H**) SW480 cell–M2 macrophage, detected by qPCR, Western blotting, and ELISA. All mRNA expression levels were calculated by the double delta CT method and normalized to the expression of GAPDH. All data were presented as the mean ± SD from at least three independent experiments. * *p* < 0.05, ** *p* < 0.01, and *** *p* < 0.001, ns present no significant, compared with the control group.

To determine whether the increased secretion of TGF-β1 was critical for the role of the increased migration, the TGF-β1-neutralizing antibody which could consume TGF-β1 in the medium was used. As a result, supplementation with a TGF-β1-neutralizing antibody in the co-culture system inhibited the migration of CRC cells, which was induced by MC-LR (Figure 3A,B), suggesting that the exposure of MC-LR enhanced the migration of CRC cells via upregulating the secretion of TGF-β1 from M2 macrophage in the co-culture system. Given that CST3 was downregulated in CRC cells in the co-culture system, whether the reduced expression of CST3 was related to CRC cell migration, we performed a transient transfection experiment to overexpress CST3 in CRC cells. Western blotting showed the overexpression of CST3 in the transfected CRC cells (Figure S3A,B). Then, we found that, compared with the control group, the overexpression of CST3 in CRC cells remarkably weakened the capacity of migration (Figure 3E,F). Furthermore, CRC cells transfected with overexpressed CST3 in the co-culture system displayed fewer migrated cells than the untransfected CRC cells (Figure 3E,F, photos 2 and 3). The promoted ability of migration induced by MC-LR was inhibited after the overexpression of CST3 in CRC cells in the co-culture system (Figure 3E,F, photos 5 and 6). Therefore, our results suggested that the reduction of CST3 in CRC cells contributed to its migration in the co-culture system.

As the predicted relationship between TGF-β1 and CST3 by IPA, to further determine whether the reduced expression of CST3 was related to the increased expression of TGF-β1, we detected the expression of CST3 by qPCR and Western blotting in CRC cells from the co-culture system under the exposure of MC-LR and supplemented with a TGF-β1-neutralizing antibody. Compared with the group treated with IgG, adding a TGF-β1-neutralizing antibody could reverse the reduced expression and secretion of CST3 caused by MC-LR in CRC cells (Figure 3C,D). After that, the influence from CST3 to TGF-β1 was also detected by qPCR and Western blotting, as shown in Figure 3G,H, in the condition of co-culture, and overexpressing CST3 in CRC cells could inhibit the mRNA and protein expression of TGF-β1 in M2 macrophages, as well as the secretion. Altogether, these results indicated that the interaction between CRC cells and M2 macrophages induced by MC-LR was achieved by increasing the expression of TGF-β1 in M2 macrophages to downregulate the expression of CST3 in CRC cells; at the same time, overexpressing CST3 could also downregulate the expression of TGF-β1 to inhibit migration. All of the above verified that MC-LR could promote the interaction in TME, which could influence each other and play a key role in promoting the migration of CRC cells.

### 2.4. MC-LR Increased M2 Macrophage Infiltration and Decreased the Expression of CST3 to Exacerbate Tumor Progression in AOM/DSS-Model Mice

**Figure 4:**
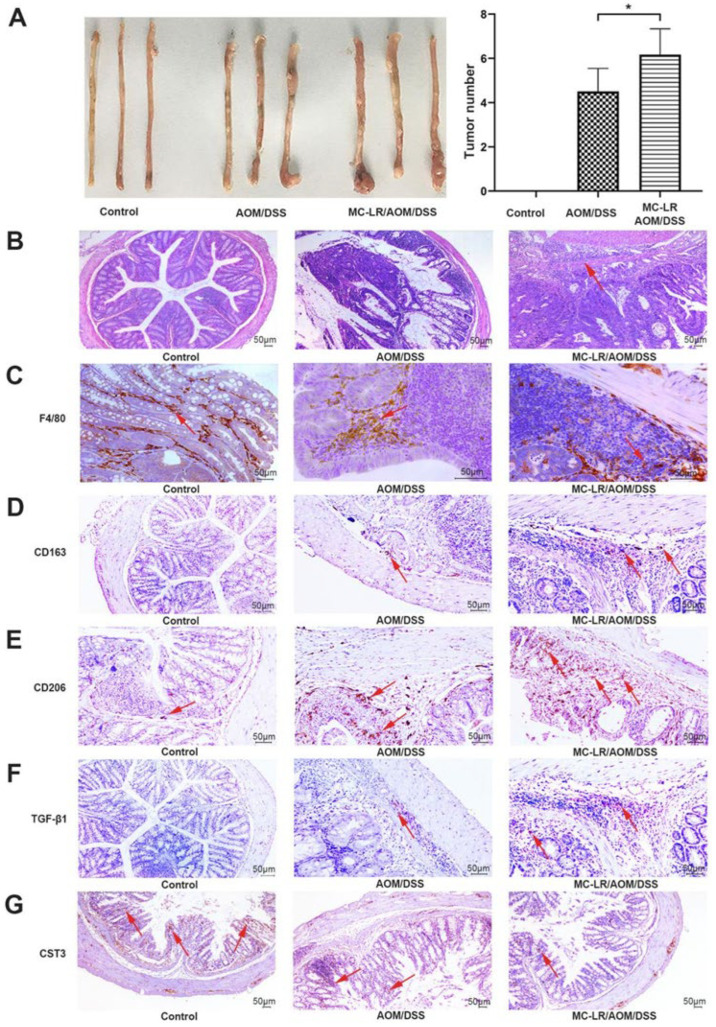
MC-LR increased M2 macrophage infiltration and decreased the expression of CST3 to exacerbate tumor progression in AOM/DSS-model mice. (**A**) Gross macroscopic images of the colorectal tissues of mice in each group were represented (*n* = 10). The number of colorectal tumors in mice was counted. (**B**) Colorectal tumor tissues of mice were stained by H&E and the arrow indicates tumor infiltration (scale bar: 50 μm). Data were presented as the means ± SD (*n* = 10). * *p* < 0.05, compared with the control group. The IHC results of CRC tissues were shown in (**C**–**F**), and (**G**), which represent the expression of F4/80, CD163, CD206, TGF-β1, and CST3, respectively, and the arrows indicated the positive expression of corresponding measurement indicators (scale bar: 50 μm). Sequential representation: the control and the AOM/DSS group were treated with saline; and MC-LR/AOM/DSS group was treated with 40 μg/kg body weight MC-LR daily for 15 days.

Given the implication of inflammation in CRC progression, the question of whether MC-LR promoted CRC progression was related to macrophages needs more comprehensive exploration as well; the AOM/DSS model in Balb/c mice, an inflammation-related colon carcinogenesis model, was constructed and the process was shown in Figure S4A. During modeling, AOM/DSS-model mice experienced obvious bloody stool and weight loss in the fourth week, whereas the control mice had no abnormalities (Figure S4B). After the success of model generation, 40 μg/kg MC-LR was administrated in MC-LR/AOM/DSS mice for 15 days, while AOM/DSS mice were given saline at the same time. Then, all mice were sacrificed and the body weight of MC-LR/AOM/DSS mice exhibited significant decrease after 1 week of MC-LR gavage compared with that of AOM/DSS mice (Figure S4B). As estimated, AOM/DSS treatment led to colorectal tumors, which were observed in the distal colon of mice by macroscopic examination, while none of the control mice formed colorectal lesions (Figure 4A). Furthermore, the number of tumor nodules in MC-LR/AOM/DSS mice was more than that in AOM/DSS mice (Figure 4A). In agreement with this, a more severe histopathology was observed in CRC tissues by H&E staining in the group of MC-LR/AOM/DSS mice, exhibiting multiple aberrant crypt foci and the tumor invaded into the deep muscle layer of CRC tissues, while the tumor of AOM/DSS mice did not infiltrate into the bowel wall and the colorectal tissues of the control mice were smooth and intact (Figure 4B).

As mentioned earlier, TAM can effectively promote tumor progression, and M2 macrophages are the core members to play their function. Subsequently, the IHC experiment demonstrated that, compared with the AOM/DSS group, the MC-LR/AOM/DSS group showed more macrophage recruitment by detecting markers of macrophages, F4/80 (Figure 4C), and more M2 macrophages infiltration in CRC tissues by detecting markers of the M2 macrophage, CD163 and CD206 (Figure 4D,E). In addition, compared with the mice of the AOM/DSS group, the level of TGF-β1 was elevated (Figure 4F) and CST3 was reduced (Figure 4G) in CRC tissues of MC-LR-treated mice, whereas the control exhibited a lower expression of TGF-β1 (Figure 4F) and higher expression of CST3 (Figure 4G) in colorectal tissues compared to that in CRC tissues of both MC-LR/AOM/DSS and AOM/DSS mice. Taken together, it is legible that MC-LR could promote the infiltration of M2 macrophages, elevate the expression of TGF-β1, and downregulate the expression of CST3 in vitro and vivo in CRC to exacerbate the progression of the tumor.

## 3. Discussion

**Figure 5:**
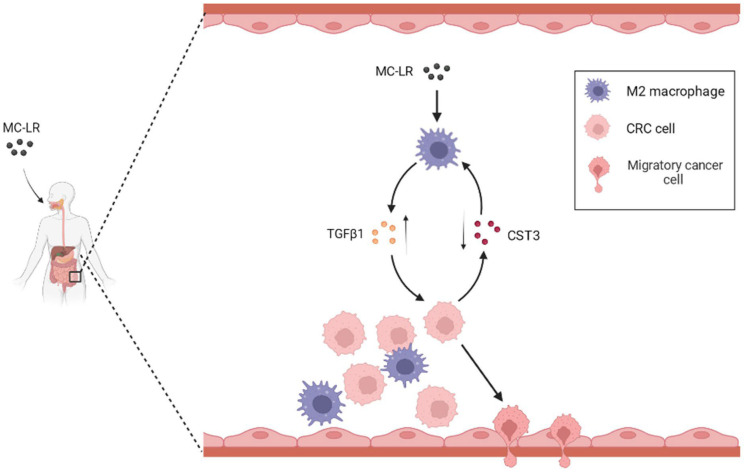
The mechanism of the promoted migration of CRC cells induced by MC-LR in the TME.

In this investigation, we unveiled a new mechanism underlying the tumor-promoting effect of MC-LR in TME and highlight the potential of targeting the MC-LR/M2-TAM/TGF-β1/CST3 axis as a therapeutic strategy for CRC (Figure 5). We discovered that MC-LR boosts the secretion of TGF-β1 by M2 macrophages in the co-culture system, thereby significantly augmenting CRC cell migration. TGF-β1 elicits its promotion by suppressing CST3 expression in CRC cells, and neutralizing TGF-β1 or overexpressing CST3 both impede the migration of CRC cells.

The gut acts as a natural barrier that maintains intestinal stability, inhibiting pathogenic bacteria and toxins. Unfortunately, the intestinal barrier is vulnerable to damage caused by MC-LR, which disrupts tight connections between epithelial cells and induces intestinal barrier leakage [27], which can lead to the development and exacerbation of gastrointestinal diseases. Our previous research [28] has demonstrated that MC-LR enhances the migration ability of CRC cells. In tumor tissue, MC-LR infiltrates the tumor microenvironment, regulating not only tumor cells but also infiltrating cells such as immune cells, fibroblasts, and endothelial cells. The symptoms observed are the result of mutual regulation among various cells. M2-TAMs are known to promote tumor growth and metastasis and are associated with a poor prognosis [29]. Therefore, we investigated the interaction between M2 macrophages and tumor cells in the context of MC-LR exposure. Our experimental results confirmed that M2 macrophages promote the migration of colorectal cancer cells under MC-LR stimulation. Furthermore, animal experiments have shown that MC-LR promotes M2-TAM infiltration, and the degree of infiltration is positively correlated with tumor progression.

The Transwell co-culture system is used to investigate intercellular communication between CRC cells and M2 macrophages, as it separates the two types of cells and, thus, their communication occurs mainly through the secretion of cytokines and chemokines. MC-LR has been shown to regulate the secretion of various cytokines and chemokines in physiological and pathological processes. For instance, it can induce human umbilical vein endothelial cells to secrete IL-1β, IL-6, and IL-8, resulting in oxidative stress and inflammation [30]. Other studies have shown that MC-LR induces the production of inflammatory cytokines and chemokines such as IL-6, MCP-1, and CXCL10, and activates innate immune responses in mouse testicular cells, leading to testicular inflammation [31]. MC-LR has also been shown to enhance TNF-α and IL-1β expression in mouse peritoneal macrophages, thus promoting M1 polarization [32]. Similarly, our study has shown that MC-LR enhanced the expression of TGF-β1 under the co-culture system. Interestingly, the upregulation of TGF-β1 was found to be caused by the secretion of M2 macrophages, rather than CRC cells. As demonstrated by Yang et al., it was essential for the upregulation of TGF-β1 in M2 macrophages under the co-culture system involving the ESSC cell and M2 macrophages [33], which is consistent with our results.

TGF-β is a multifunctional cytokine that comes in three subtypes, with TGF-β1 being the most common [34]. It plays vital roles in growth and development, inflammation repair, and host immunity [35]. In the context of tumor development, TGF-β has a dual role, acting as a tumor suppressor in premalignant cells but as a tumor promoter in malignant cells [36,37]. While TGF-β exerts inhibitory pressure on precancerous cells, enabling clones of cancer cells to escape its tumor-suppressive effects through TGF-β pathway inactivation or dissociation, it also promotes invasion and immune evasion, converting TGF-β from a suppressive obstacle to a metastasis stimulant [38]. Numerous studies have found that the increased expression of TGF-β is associated with tumor progression and a poor prognosis [39]. In particular, the study by Qin and Shen et al. confirmed the high expression of TGF-β in CRC tissue, which is one of the reasons for the occurrence and development of colorectal cancer [40,41]. Consistently, our animal experiments demonstrated a high expression of TGF-β1 in tumor tissues induced by MC-LR-stimulated M2-TAM. Additionally, the addition of a TGF-β1-neutralizing antibody in the co-culture system significantly reduced the migration ability of CRC cells, suggesting that the increased secretion of TGF-β1 in the supernatant was responsible for the enhanced migration ability of CRC cells. Further investigation revealed that the tumor suppressor gene CST3 in CRC cells was also involved in CRC cell migration and was regulated by TGF-β1.

CST3, a member of the cystatins family, is known to inhibit cathepsins, especially cathepsin D [42], and regulate various physiological and pathological processes [43]. Recent studies have shown that CST3 expression negatively correlates with cancer development in breast cancer [44,45], CRC [46], bladder cancer [47], and hepatocellular carcinoma [48]. In this study, we demonstrate the inhibitory role of CST3 in CRC cell migration and provide evidence for a negative regulatory relationship between TGF-β1 and CST3. Firstly, we added a TGF-β1-neutralizing antibody to the co-culture medium, which significantly decreased the CRC cell migration ability and increased the expression of CST3 in CRC cells. Secondly, we constructed a CRC cell line that overexpressed CST3, which inhibited the expression of TGF-β1 in M2 macrophages and significantly weakened the CRC cell migration ability.

While we have discovered a novel pathway whereby MC-LR impacts the interaction between M2 macrophages and CRC cells within the tumor microenvironment, additional research is necessary to comprehensively elucidate the mechanism by which TGF-β1, derived from M2 macrophages stimulated by MC-LR exposure, impedes CST3 expression in CRC cells, further facilitating CRC cell migration. Several reports have indicated that the expression of the transcript of CST3 can be regulated by TGF-β1 [49,50,51], but the specific mechanism of regulation has not been fully elucidated. Previous studies have suggested that TGF-β1 can promote glycolysis and L-lactate production while inhibiting ATPase activity in renal cells, potentially indicating a relationship between TGF-β1 and the tumorous acidic environment [52,53]. In addition, recent studies have shown that cathepsin D can cleave CST3 under an acidic pH, leading to decreased CST3 expression in the breast cancer microenvironment [54,55]. Therefore, we hypothesize that the reduced CST3 expression observed in our study may be associated with cathepsin D and the acidic microenvironment. Specifically, the high expression of TGF-β1 induced by MC-LR exposure in M2 macrophages may stimulate glycolysis and L-lactate production in CRC cells, leading to an acidic microenvironment that activates cathepsin D and enhances the cleavage of CST3, ultimately leading to reduced CST3 expression. In addition, prior research has indicated that CST3 can interact with the TGF-β type II receptor to prevent the binding of TGF-β1 to the receptor, thereby inhibiting TGF-β1 signaling [49]. However, additional investigation is required to fully elucidate the precise mechanisms underlying the impact of MC-LR on the interplay between M2 macrophages and tumor cells within the tumor microenvironment, as well as its role in regulating CST3 expression in tumor cells and subsequent effects on the expression of TGF-β1 in M2 macrophages.

Overall, our study has uncovered a crucial mechanism underlying the promotion of CRC cell migration by MC-LR in the TME. We found that MC-LR can upregulate the expression and secretion of TGF-β1 in M2 macrophages in the co-culture system, which, in turn, downregulates the expression of CST3 in CRC cells, which is also confirmed by animal experiments. The combined effect of MC-LR-regulated TGF-β1 and CST3 facilitates the migration of CRC cells. Importantly, neutralizing TGF-β1 in the culture medium or overexpressing CST3 in CRC cells effectively reduced the migration ability of CRC cells. In summary, our study not only sheds light on the possible mechanism of MC-LR, but also provides potential strategies for controlling and treating colorectal cancer by modulating the tumor microenvironment.

## 4. Materials and Methods

### 4.1. Cell Culture and THP-1 Polarization

Human colorectal cancer cell lines (DLD-1; SW480) were obtained from the Type Culture Collection of the Chinese Academy of Sciences. CRC cells were cultured in Dulbecco’s modified Eagle’s medium (DMEM) (Gibco, Billings, MT, USA) supplemented with 10% fetal bovine serum (FBS) (Lonsa, Uruguay, South America), 100 units/mL streptomycin, and 100 units/mL penicillin (Thermo, Waltham, MA, USA) in a humidified incubator (5% CO_2_, 37 °C).

Human monocytes THP-1 cells which were obtained from the Type Culture Collection of the Chinese Academy of Sciences were cultured at 37 °C in a humidified 5% CO_2_ atmosphere. THP-1 cells were stimulated with 100 nM phorbol 12-myristate 13-acetate (PMA) (Sigma-Aldrich, St. Louis, MO, USA) for 48 h to allow them to attach and differentiate into M0 macrophages. Next, M0 macrophages were treated with 20 ng/mL IL-4 (Peprotech, Cranbury, NJ, USA) and 20 ng/mL IL-13 (Peprotech, US) cytokines for 48 h to polarize to M2 macrophages. To detect the effects of MC-LR (purity ≥ 95%, ENZO, Broomfield, CO, USA) or CRC cells on M2 macrophages, 25 nM MC-LR or supernatants of CRC cells pretreated with or without 25 nM MC-LR for 48 h were cultivated with M2 macrophages.

### 4.2. Co-Culture of CRC Cells and M2 Macrophages

CRC cells and M2 macrophages were co-cultured using a 6-well Transwell chamber with a 0.4 μm porous membrane (Corning, New York, NY, USA), which separated the DLD-1 or SW480 cells (5.0 × 10^5^ cells/well) in the upper chamber from the M2 macrophages (2.0 × 10^6^ cells/well) in the lower chamber. To analyze the effects of MC-LR on M2 macrophages and CRC cells, the co-culture system was treated with 25 nM MC-LR for 48 h. Then, the cells and supernatants were harvested separately for subsequent experiments.

### 4.3. Animal Experiments

Male Balb/c mice (18–20 g) were obtained from the Institute of Model Animals of Nanjing University (Nanjing, China), and the mice were bred and maintained in a standardized environment for one week. After the acclimation period, the mice were randomly divided into three groups: control, AOM/DSS model establishment, and MC-LR exposure on AOM/DSS model (ten mice in each group). The mice of the AOM/DSS model group were intraperitoneally injected with 12.5 mg/kg body weight azoxymethane (AOM, Sigma, St. Louis, MO, USA) on the first day of model establishment. One week later, the mice were given 2.5% DSS in the drinking water for one week, followed by two weeks of regular drinking water. The cycle of water contained 2.5% DSS and regular water was repeated six times. After the establishment of the AOM/DSS model, AOM/DSS mice were given 40 μg/kg body weight MC-LR or saline via gastric intubation daily for 15 days, while the mice of the control group were given saline. We measured body weight once a week until the termination of the experiment. After the treatment of MC-LR, all mice were sacrificed, and the colorectal tissues of mice were collected, flushed with PBS, and cut longitudinally. The tumor numbers of mice in each group were quantified based on gross examinations. Colorectal samples were fixed in 10% buffered formalin (pH 7.4), and remaining samples were frozen in liquid nitrogen for further RNA and protein analysis. All animal studies were monitored and sanctioned by the Guide for the Care and Use of Laboratory Animals by the Medical Experimental Animal Care Commission of Nanjing Medical University.

### 4.4. Flow Cytometry

THP-1 cells induced by PMA, IL4, and IL13, successively, were harvested and digested in trypsin-EDTA solution at 37 °C for 5–10 min. Subsequently, the cells were washed three times in FACS buffer (2% FBS-PBS) and centrifuged at 1000× *g* for 5 min. Next, the cells were stained with fluorescently labeled antibodies, CD163 (326505, Biolegend, San Diego, CA, USA) or CD206 (321109, Biolegend, US), at 4 °C for 20 min in dark. After washing and resuspending in FACS buffer, the cells were analyzed by a flow cytometer (BD Bioscience, San Jose, CA, USA).

### 4.5. Western Blotting

CRC cells and macrophages were lysed by the RIPA solution to extract the protein (Thermo Fisher Scientific, Waltham, MA, USA), then the protein concentration was measured with the BCA Protein Assays Kit (Beyotime, Shanghai, China). The proteins were separated by SDS-PAGE and transferred to polyvinylidene fluoride (PVDF) membrane, which was then blocked for 2 h at room temperature, and subsequently incubated with primary antibodies, including anti-GAPDH (5174, 1:1000, Cell Signal Technology, Danvers, MA, USA), anti-TGF-β1 (ab215715, 1:1000, Abcam, Cambridge, UK), and anti-CST3 (ab109508, 1:1000, Abcam, UK) overnight at 4 °C. Next, membranes were incubated with the appropriate horseradish peroxidase-conjugated secondary antibodies (7076, 1:1000, Cell Signal Technology, Danvers, MA, USA) for 2 h at room temperature. Blots were visualized by enhanced chemiluminescence on a Tanon 5200 system (Tanon, Shanghai, China).

### 4.6. Quantitative Real-Time Polymerase Chain Reaction

Total RNA from CRC tissues, CRC cells, or M2 macrophages were extracted using the RNAiso Plus reagent (TaKaRa, Osaka, Japan) followed by reverse transcription to cDNA, and then qPCR was performed based on SYBR green (SYBR green qPCR Master Mix; Vazyme, Nanjing, China). The reaction system includes 10 μL 2 × ChamQ SYBR qPCR Master Mix, 0.4 μL 50 × ROX Reference Dye2, 0.5 μL primer mix, 1 μL template cDNA, and make up with nuclease-free water to a final volume of 20 μL. The amplification conditions were 95 °C for 35 s, 40 cycles of 95 °C for 10 s, and 60 °C for 30 s. All reactions were run in triplicate using an ABI 7500 PCR System (Applied Biosystems, Waltham, MA, USA). mRNA expression levels of target gene expression were normalized to GAPDH levels using the formula 2^−ΔΔCT^. These sequences of used primers are shown in Table S1.

### 4.7. Enzyme-Linked Immunosorbent Assay (ELISA)

TGF-β1 and CST3 in the supernatant harvested from the co-culture system (see co-culture of CRC cells and M2 macrophages) were quantified by using the Human TGF-β1 ELISA kit and CST3 ELISA kit (MultiSciences, Hangzhou, China) according to the manufacturer’s instructions. All samples were analyzed in triplicate.

### 4.8. Transfection In Vitro

CST3 plasmid or empty plasmid was, respectively, transfected into DLD-1 and SW480 cells at 50% confluence using Lipofectamine 3000 (Life Technologies, Carlsbad, CA, USA) according to the manufacturer’s instructions. Then, the transfected cells were collected to co-culture with M2 macrophages or conduct Transwell migration assays.

### 4.9. Transwell Migration Assay

Cell migration assays were performed using Transwell chambers (24-wells, 8 µm, Corning, US). The surface of the upper chamber was plated with CRC cells or overexpression CST3 CRC cells at 5 × 10^4^ per well in 200 μL of serum-free media (Gibco) or in 25 nM MC-LR treated serum-free media. The lower chamber was plated with M2 macrophages at 1 × 10^6^ per well in 550 μL of complete medium or complete medium alone. In addition, for the co-culture system, 0.8 μg/mL TGF-β1-neutralizing antibody (R&D Systems, Minneapolis, MN, USA) or IgG (Beyotime Biotechnology, Shanghai, China) was added to determine the effects of TGF-β1 on CRC cell migration. After 48 h, the chambers were fixed with 4% paraformaldehyde and stained with 0.5% crystal violet before the cells on the upper surface were removed completely with a cotton swab. The translocated cells were observed under light microscopy at a magnification of ×40.

### 4.10. Hematoxylin & Eosin (H&E) Staining

Colorectal tissues from all mice were freshly collected and gently washed with PBS, and full rolls were placed before fixation in 10% neutral buffered formalin and paraffin embedding. Sections were stained with hematoxylin for 5 min after deparaffinization and rehydration, and immersed in 1% acid ethanol. Then, the eosin solution was used to stain sections for 3 min, and sections were immersed in alcohol and xylene to dehydrate and clear. Finally, slides were mounted and imaged under a Leica microscope (Leica, Wetzlar, Germany).

### 4.11. Immunohistochemistry (IHC)

Colorectal tissues were gently washed with PBS, and full rolls were placed before fixation in 10% neutral buffered formalin and paraffin embedding. Sections were deparaffinized in xylene and rehydrated before heated antigen retrieval in 10 mM sodium citrate buffer (pH 6.0). Then, sections were covered and incubated with appropriate antibodies (anti-TGF-β1 (ab215715, Abcam, UK), anti-CST3 (ab109508, Abcam, UK), anti-CD163 (ab182422, Abcam, UK), and anti-CD206 (ab64693, Abcam, UK)), at 4 °C for 24 h. Subsequently, the hematoxylin staining was applied before staining with diaminobenzidine (DBA). Finally, sections were visualized using a Leica microscope.

### 4.12. Isobaric Tags for Relative and Absolute Quantification (iTRAQ)

Secretion protein samples harvested from the co-culture system were prepared for iTRAQ labeling and mass spectrometry, and streptavidin beads were included to enrich secreted proteins from the cell supernatant. Subsequently, on-bead digestion was performed to retrieve peptides. After iTRAQ labeling of digested peptides, strong cation exchange (SCX) was used to separate labeled peptides, and bRPLC fractionation was performed followed by protein identification and quantitative proteomics data analysis. Different proteins expressed under the conditions of the MC-LR treatment or not were presented as fold change (FC), and FC ≥ 1.2 means differential expression. To explore the potential functions of differentially expressed proteins and the pathways that these proteins might be involved in, gene ontology (GO (https://david.ncifcrf.gov (accessed on 11 July 2019))) analysis was performed including cellular component, molecular function, and biological process. Moreover, ingenuity pathway analysis (IPA) was used to study the factors underlying the relationship between screened proteins and advanced malignant tumors. The iTRAQ data are listed in Table S1.

### 4.13. Statistical Analysis

Statistical analyses were performed by using GraphPad 8.0 (San Diego, CA, USA) and data were presented as the mean ± standard deviation (SD). One-way ANOVA was used to compare two groups followed by Student’s *t*-test. *p* < 0.05 was identified as statistical difference.

## 5. Conclusions

Taken together, the key findings of our study included that MC-LR was involved in the interaction between CRC cells and M2 macrophages in TME, where MC-LR increased the secretion of TGF-β1 from M2 macrophages into TME. Meanwhile, the upregulation of TGF-β1 in TME could reduce the expression of CST3 in CRC cells, further resulting in the migration of CRC cells. These findings shed new light on the role of MC-LR in the TME of CRC, providing a mechanistic basis for CRC cell migration promoted by MC-LR and a potential strategy to inhibit CRC progression and metastasis.

## Data Availability

All data generated during this study are included in this published article (and its Supplementary Information files) or are available from the corresponding author upon reasonable request.

## Supplementary Materials

The supporting information can be downloaded at: https://www.mdpi.com/article/10.3390/ijms241310527/s1.
