# Supplementary material for: Microcystin-LR-Induced Interaction between M2 Tumor-Associated Macrophage and Colorectal Cancer Cell Promotes Colorectal Cancer Cell Migration through Regulating the Expression of TGF-β1 and CST3
Source: Int J Mol Sci. 2023 Jun 23;24(13):10527. doi: 10.3390/ijms241310527 (PMC10341441; doi:10.3390/ijms241310527)
Supplement: Supplementary file 1 [file ijms-24-10527-s001.zip › ijms-2420819-supplementary.pdf]

Supplementary materials

Microcystin-LR-induced interaction between M2 tumor-associated macrophage and colorectal cancer cell promotes colorectal cancer cell migration through regulating the expression of TGF-β1 and CST3

Xinying Jiang[a, †], Hailing Zhang[a, 1, †], Hengshuo Zhang[a], Fan Wang[a], Xiaochang Wang[a], Tong Ding[a], Xuxiang Zhang[b, *], and Ting Wang[a, *]

[a] Department of Cell Biology, School of Basic Medical Sciences, Nanjing Medical University, 101 Longmian Avenue, Nanjing, 211166, China
[b] State Key Laboratory of Pollution Control and Resource Reuse, School of the Environment, Xianlin Campus, Nanjing University, 163 Xianlin Avenue, Nanjing 210023, China
[1] Present affiliation: Department of Biochemistry, Medical College, Anhui Medical University, 1166 Wangjiang West Road, Hefei, Anhui 230031, China
[†] These authors contributed equally to this work

***Correspondence authors:**

Xuxiang Zhang, PhD

NJU, School of the Environment

Av. Xianlin, 163

210023-Nanjing, China

Ting Wang, PhD

NJMU, Department of Cell Biology

Av. Longmian, 101

211166-Nanjing, China

Table S1. Primers used for qPCR analysis

Fig. S1. Effects of MC-LR exposure on M2 macrophages

Fig. S2. GO enrichment analysis of differential expression proteins

Fig. S3. Verification of CST3 in CST3-overexpression CRC cells and the effect of IgG in the co-culture system treated with MC-LR.

Fig. S4. Effect of MC-LR on AOM/DSS mouse model

Table S1. Primers used for qPCR analysis

Gene name	Primer sequence (5`-3`)
TGFβ1	Forward: CTAATGGTGGAAACCCACAACG<br>Reverse: TATCGCCAGGAATTGTTGCTG
TGFβ1 (mus)	Forward: TGCTAATGGTGGACCGCAA<br>Reverse: CACTGCTTCCCGAATGTCTGA
CST3	Forward: GATCGTAGCTGGGGTGAACT<br>Reverse: CCTTTTCAGATGTGGCTGGT
CST3 (mus)	Forward: CAAAACAAGGCCCGCGAAT<br>Reverse: GGAGCAGAGTGCCTTCCTCA
IL10	Forward: GACTTTAAGGGTTACCTGGGTTG<br>Reverse: TCACATGCGCCTTGATGTCTG
Arg-1	Forward: CTGTGGGAAAAGCAAGCGAG<br>Reverse: CATGGCCAGAGATGCTTCCA
CD206	Forward: CTACTGAACCCCCACAAC<br>Reverse: AAACCAGAGAGGAACCCA
GAPDH	Forward: CACCATCTTCCAGGAGCGAG<br>Reverse: GATGGCATGGACTGTGGTCA

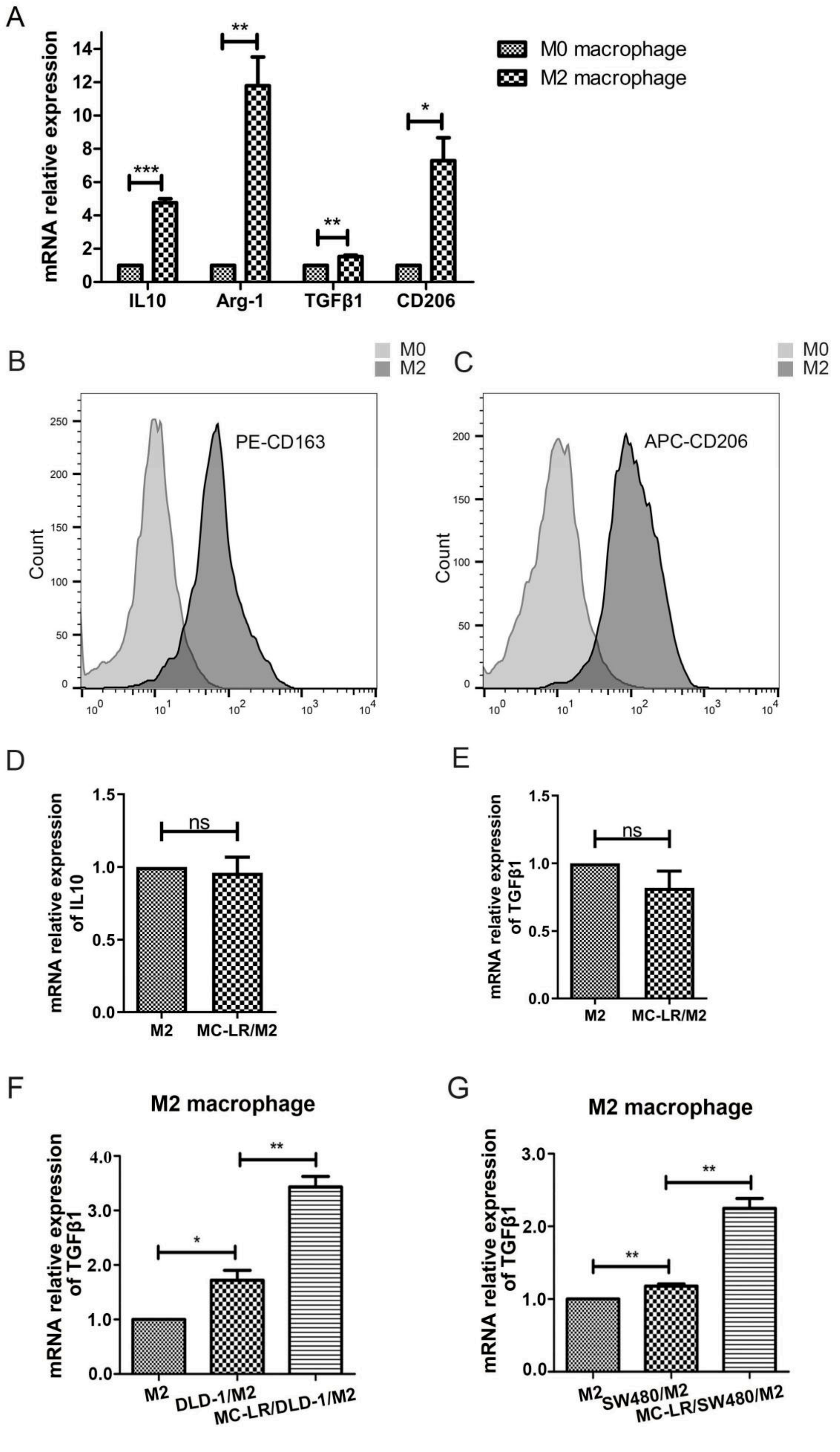
A
mRNA relative expression
M0 macrophage
M2 macrophage
IL10
Arg-1
TGFβ1
CD206
B
M0
M2
PE-CD163
Count
C
APC-CD206
D
mRNA relative expression of IL10
ns
M2
MC-LR/M2
E
mRNA relative expression of TGFβ1
F
M2 macrophage
DLD-1/M2
MC-LR/DLD-1/M2
G
SW480/M2
MC-LR/SW480/M2

12

Fig. S1. Effects of MC-LR exposure on M2 macrophages. (A) mRNA expression levels of IL-10, Arg-1, TGF-β1 and CD206 evaluated by qPCR in M0 and M2 macrophages. Positive M2 macrophages ratio labeled by (B) PE-CD163 and (C) APC-CD206 analyzed by flow cytometry. mRNA expression levels of (D) IL-10 and (E) TGF-β1 analyzed by qPCR in MC-LR-treated M2 macrophages. M2 macrophages were treated with or without 25 nM MC-LR for 48 h. mRNA expression levels of TGF-β1 in M2 macrophages, which were treated with or without (F) DLD-1 supernatant and (G) SW480 supernatant in the presence or absence of 25 nM MC-LR for 48 h, were detected by qPCR. mRNA expression was calculated by the double delta CT method ($n = 3$), normalized to GAPDH. * $P < 0.05$, ** $P < 0.01$, *** $P < 0.001$, ns means no significance compared with the control group.

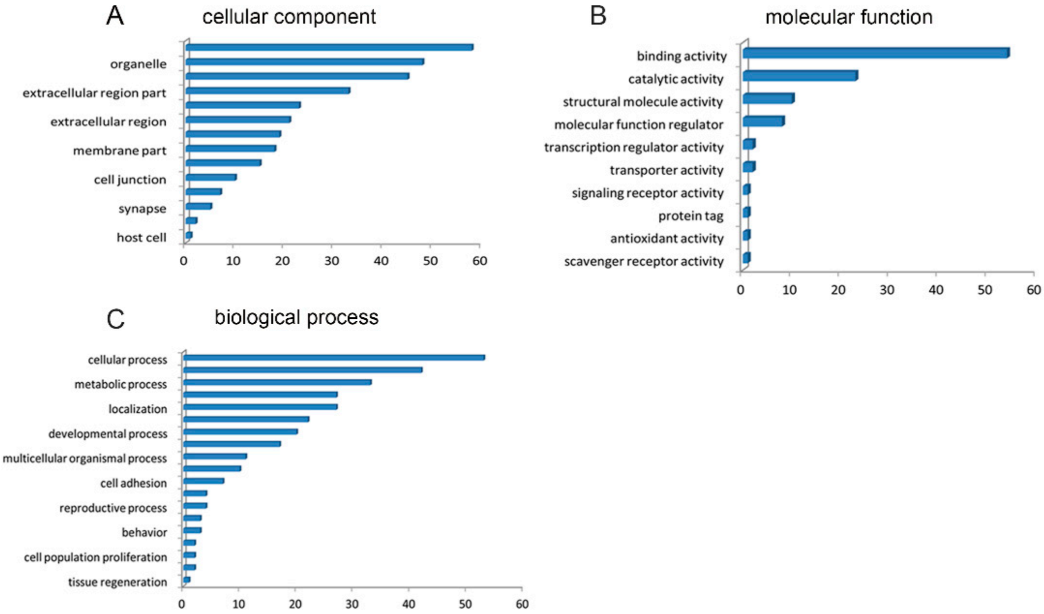

Fig. S2. GO enrichment analysis of differential expression proteins. (A) cellular component, (B) molecular function and (C) biological process.

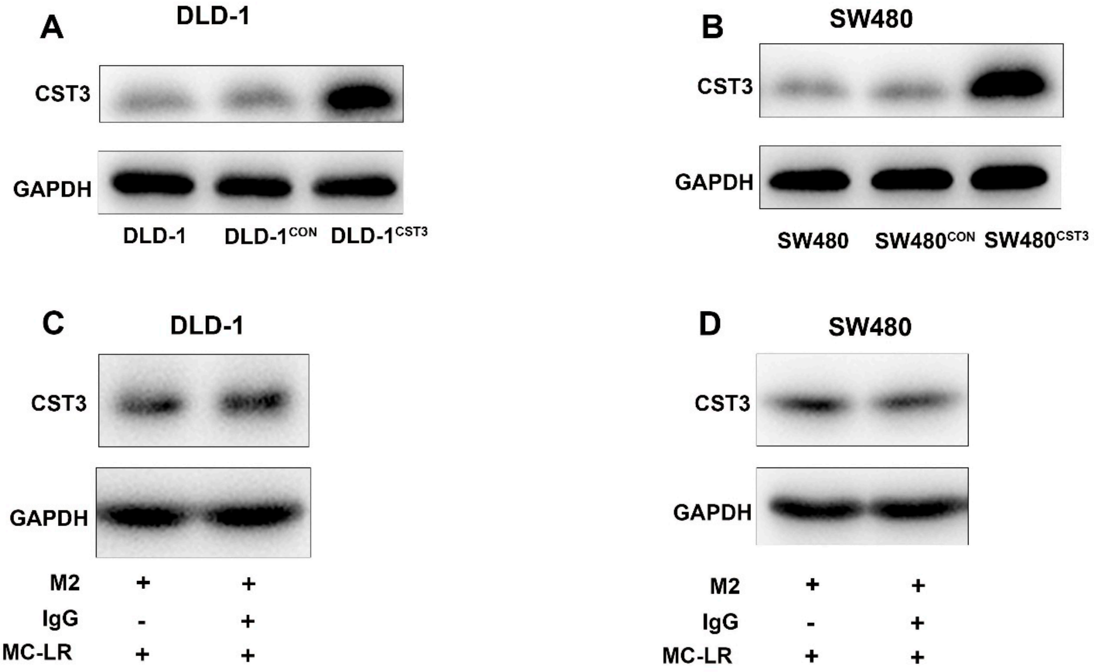

Fig. S3. Verification of CST3 in CST3-overexpression CRC cells and the effect of IgG in the co-culture system treated with MC-LR. DLD-1 cells and SW480 cells were transfected with CST3-overexpression vectors, using non-transfected cell lines and blank vectors transfected cell lines as controls. Protein expression of CST3 in CST3-overexpressing (A) DLD-1 cell and (B) SW480 cell was detected by Western blotting, respectively. Western blotting was performed to verify the protein expression of CST3 in (C) DLD-1 cell and (D) SW480 cell -M2 macrophage co-culture systems which were treated with 25 nM MC-LR with or without IgG for 48 h, isotype control of TGF-β1-neutralilizing antibody for 48 h, respectively.

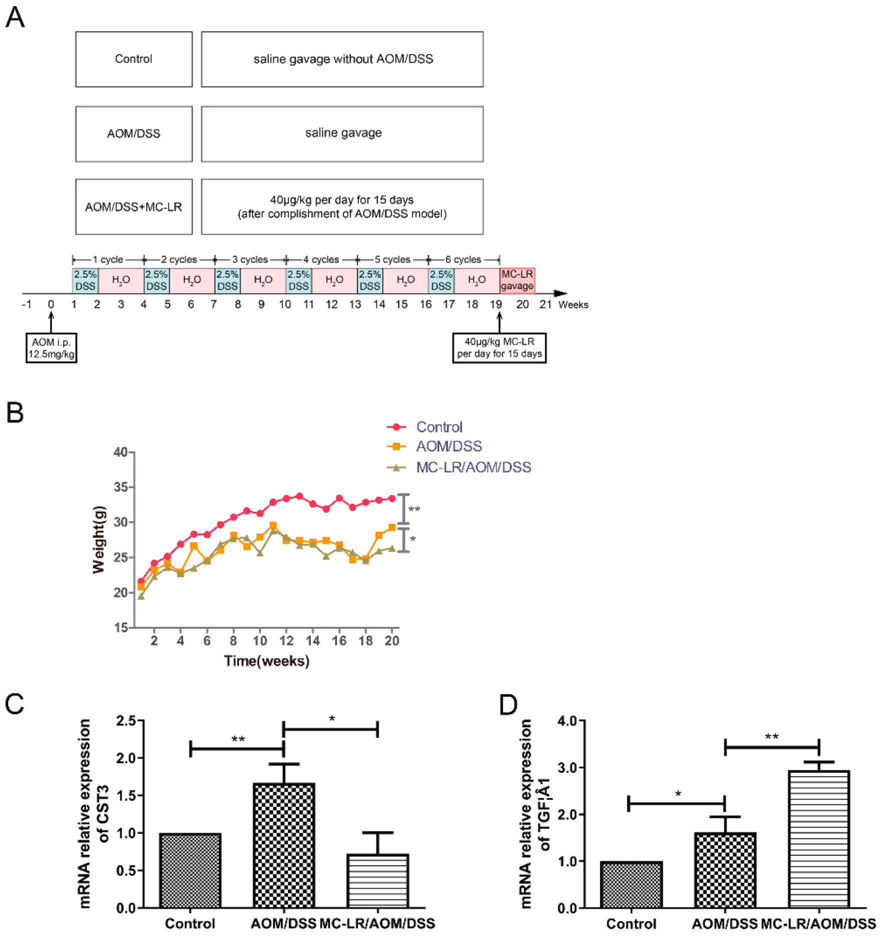

Fig. S4. Effect of MC-LR on AOM/DSS mouse model. (A) The AOM/DSS mice were treated with 40 μg/kg body weight MC-LR or saline, while the control mice were treated with saline daily for 15 days after AOM/DSS mouse model was established. (B) Body weight of mice was measured (n=10). mRNA expression levels of (C) CST3 and (D) TGF-β1 evaluated by qPCR in CRC tissues. All mRNA expression levels were calculated by the double delta CT method and normalized to the expression of GAPDH. All data was presented as the mean ± SD from at least three independent experiments. * $P < 0.05$, ** $P < 0.01$, compared with the control group.
